# Spin Labeling of Surface Cysteines Using a Bromoacrylaldehyde Spin Label

**DOI:** 10.1007/s00723-021-01350-1

**Published:** 2021-06-10

**Authors:** Graham Heaven, Michael A. Hollas, Lydia Tabernero, Alistair J. Fielding

**Affiliations:** 1 Department of Chemistry, The University of Manchester, Manchester, M13 9PL UK; 2 School of Biological Sciences, Faculty of Biology Medicine and Health, University of Manchester, Manchester, M13 9PL UK; 3 Centre for Natural Products Discovery, School of Pharmacy and Biomolecular Sciences, Liverpool John Moores University, Liverpool, L3 3AF UK

## Abstract

Structural investigations of proteins and their biological complexes are now frequently complemented by distance constraints between spin labeled cysteines generated using double electron–electron resonance (DEER) spectroscopy, via site directed spin labeling (SDSL). Methanethiosulfonate spin label (MTSSL), has become ubiquitous in the SDSL of proteins, however, has limitations owing to its high number of rotamers, and reducibility. In this article we introduce the use of bromoacrylaldehyde spin label (BASL) as a cysteine spin label, demonstrating an advantage over MTSSL due to its increased selectivity for surface cysteines, eliminating the need to ‘knock out’ superfluous cysteine residues. Applied to the multidomain protein, His domain protein tyrosine phosphatase (HD-PTP), we show that BASL can be easily added in excess with selective labeling, whereas MTSSL causes protein precipitation. Furthermore, using DEER, we were able to measure a single cysteine pair distance in a three cysteine domain within HD-PTP. The label has a further advantage of comprising a sulfide in a three-bond tether, making it a candidate for protein binding and in-cell studies.

**Supplementary Information:**

The online version contains supplementary material available at 10.1007/s00723-021-01350-1.

## Introduction

Modern studies in structural biology are facing increasingly complex systems and a range of techniques are required that can study not only small crystallisable subunits, but also large multidomain proteins and their complexes. Double electron–electron resonance (DEER) spectroscopy [1–3] has proven to be an excellent addition to the structural biologist’s toolkit. It is commonly used to extract distance constraints, typically in the range of 17–80 Å, between unpaired electrons in spin labeled cysteine residues, by measuring their electron–electron dipolar coupling interaction [4]. This is a powerful method for investigating conformational changes of proteins as well as in the presence of binding partners in solution [5].

One of the key advantages of using DEER in biological studies is that it is selective for the radical signal regardless of its environment and protein size, provided that the radical probe is in high enough concentration to measure and is not rapidly reduced by its environment. This contrasts with nuclear magnetic resonance (NMR) studies where the protein size poses a much greater challenge due to spectral crowding and line broadening [6, 7]. However, with increased protein size comes an increased likelihood of cysteine residues, which require more rounds of mutagenesis potentially making this spin labeling approach prohibitive in the case of large protein complexes and the need for alternative labeling methods such the use of a tyrosine residue [8] or the genetic incorporation of an unnatural amino acid [9].

**Fig. 1:**
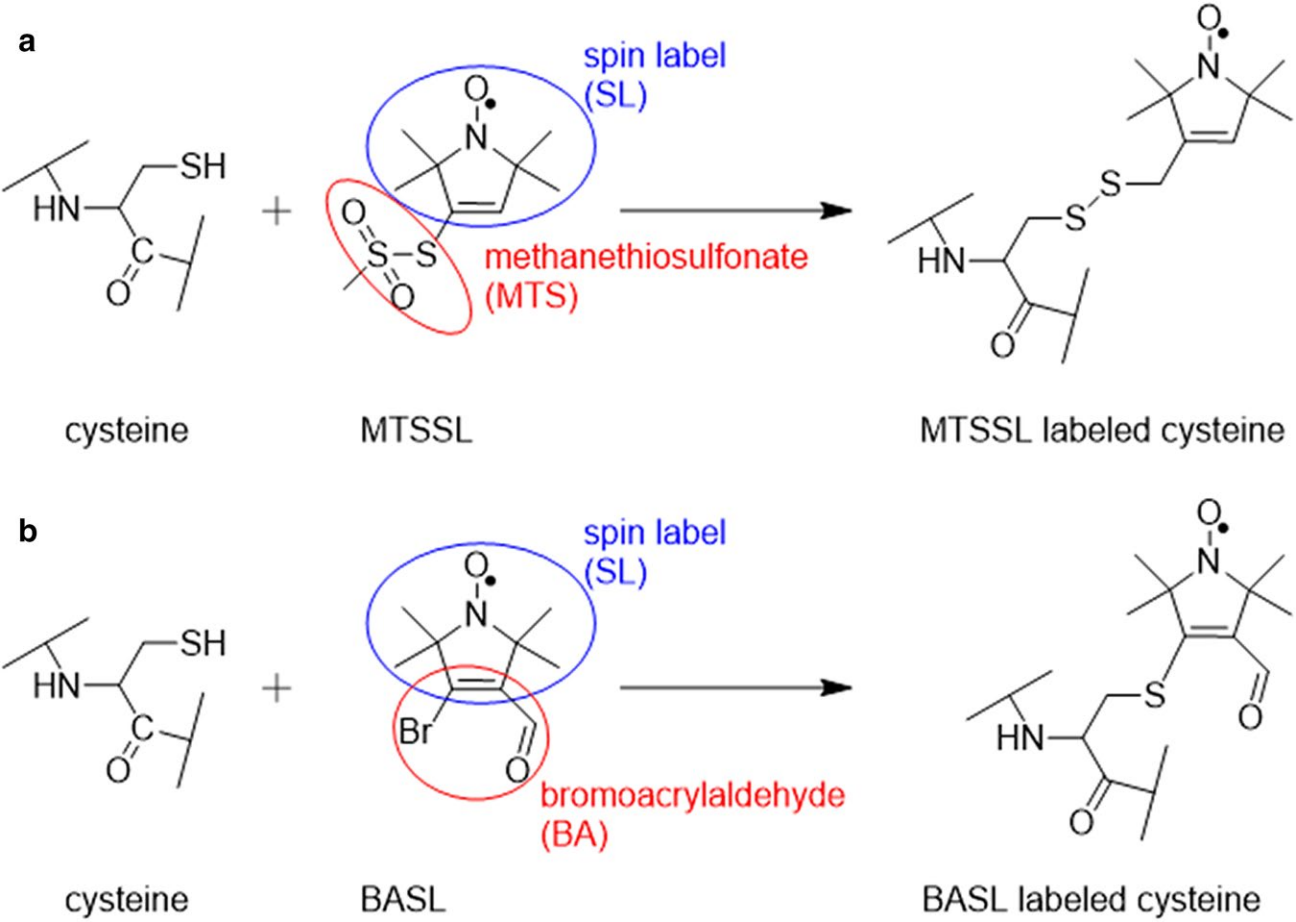
Comparison of cysteine spin labels **a** MTSSL and **b** BASL

The most widely used spin label to label proteins is the methanethiosulfonate spin label (MTSSL, Fig. 1a) [10]; highly selective for cysteine residues, with well-documented rotamer and internal side chain dynamics [11–16]. Site directed spin labeling (SDSL) studies are usually carried out between two labels on proteins whose 3D structures are known by crystallography, and labeling sites are chosen which are exposed to the surface. These surface cysteines are preferred because the resulting modified side chains are less likely to perturb structure and MTSSL can sample more easily predictable rotamers, allowing more accurate simulations of DEER distance distributions. Alternatively, it has also been shown that distance constraints can be obtained from multi-spin labeled proteins. The presence of more than two spin labels can lead to signal contributions from sum and difference combinations of the dipolar frequencies and the appearance of “ghost” peaks in distance distributions [17], however, experimental and analytical methods have been developed to identify and reduce these effects in homomultimers, demonstrated using model compounds and proteins [17–20].

There are many alternative protein spin labels to MTSSL, which have been investigated for their various properties including radical stability, linker length, and flexibility [21–33]. The choice of spin label grows ever more important with the increasing number of investigations inside cells [34]. One bifunctional nitroxide that has proven thiol reactivity is 4-bromo-3-formyl-2,2,5,5-tetramethyl-2,5-dihydro-1H-pyrrol-1-yloxyl radical (Fig. 1b). This compound was originally synthesized by Chudinov et al. [35]. It has the same 5-membered pyrroline ring nitroxide as MTSSL but instead of a methanethiosulfonate (MTS) group it has a bromine at position 4 and a formaldehyde group at position 3 of the ring. Separated by a double bond these two groups can be described as a bromoacrylaldehyde (BA) group so this compound is referred to as bromoacrylaldehyde spin label (BASL). Interestingly, compared to MTSSL, BASL will have different chemical reactivity and produce a protein label with a sulfide link and three rotatable bonds to the protein side chain as opposed to a five bond disulfide linker (Fig. 1a).

BASL has been recently used for spin labeling of bioactive ligands on gold nanoparticles [36]. Utilizing the bifunctional BA group, allowed reaction with a thiol by nucleophilic substitution of the bromine and reaction with an amine by reductive amination of the aldehyde. Previous studies by Hideg et al. have shown that BASL reacts to both the thiol and amine ends of cysteamine causing it not to be regarded as a selective spin label reagent [37]. However, we observed that the reaction of thiols with the spin label was significantly faster than the reaction with amines; which we believe were substantially slowed by steric hindrance. It was theorized under the right reaction conditions, it was possible to avoid the coupling of BASL to available lysine residues.

Previously DEER was used to investigate the structure of the multidomain phosphatase HD-PTP (His domain protein tyrosine phosphatase) [38]. HD-PTP interacts with several endosomal sorting complexes required for transport (ESCRTs) to mediate intraluminal vesicle formation at the endosome [39–42]. The three native cysteines of HD-PTP_CC_ domain were labeled by MTSSL and individual distance measurements were determined by single cysteine to serine substitutions, and used to probe the structure of coiled-coil (CC) domain [38].

In this work, it is shown for the first time that BASL can be used to successfully label cysteine residues in proteins allowing DEER measurements to take place. Using HD-PTP_CC_ and HD-PTP_Bro1_, BASL was found to be significantly more selective for surface cysteine residues compared to MTSSL.

## Materials and Methods

### Purification of HD-PTP

Cell pellets (~ 1.5 g after expression by IPTG induction in LB) were resuspended in 20 mM HEPES pH 7.4, 500 mM NaCl, 2 mM DTT, 0.1% Triton x-100, 0.5 mg/mL lysozyme, and a complete protease inhibitor tablet. For lysis, cells were sonicated 8 × 30 s on, 30 s off at 30% amplitude. After centrifugation at 13,000 rpm lysates were purified by nickel affinity chromatography [38, 43].

### MTSSL Labeling

Nickel column elution fractions were combined and 5 mM EDTA and 10 mM DTT were added before concentrating to 250 μL and loading onto a Superdex200 column equilibrated in 20 mM HEPES pH 7.4, 250 mM NaCl, 2 mM EDTA. This first size exclusion removed DTT from the sample, allowing the cysteines to be labeled. Labeling was carried out in the presence of various amounts of the spin label MTSSL (1–15 × molar excess) for 1 h at 4 ℃ with gentle rocking and rolling. After this, the sample was concentrated again for size exclusion to remove excess spin label.

### BASL Labeling

Protein fractions from the nickel column were combined and 5 mM EDTA and 10 mM DTT were added as before but instead the Superdex200 was equilibrated in 20 mM Tris pH 8.5, 250 mM NaCl, 2 mM EDTA. The more alkaline value of pH was thought to increase the reactivity of the Michael addition. Proteins were labeled with 15 × excess of BASL overnight 4 ℃ with gentle rocking and rolling. Size exclusion was used to remove excess spin label.

### DEER Sample Preparation

After size exclusion, the buffer was exchanged into deuterated buffer (20 mM HEPES, 250 mM NaCl, pD 7.4 = pH 7.0 using a standard pH probe). DEER samples were prepared with 30% (v/v) glycerol-d8 to a final protein concentration of 60 μM. 120 μL samples were frozen inside 4 mm quartz tubes (Wilmad) by flash freezing with liquid nitrogen, and stored in a liquid nitrogen dewar until measurement.

### Continuous-Wave EPR

Continuous-wave (CW) EPR samples were prepared in capillary tubes inserted inside 4 mm quartz tubes (Wilmad). CW EPR spectra were recorded on a Bruker MicroEMX (9 GHz, “X-band”) with a sweep width of 150 G, power attenuation of 2 mW, field modulation of 100 kHz, and modulation amplitude of 2 G.

### Mass Spectrometry

HD-PTP_CC_-MTSSL mass spectra were recorded on an Agilent 6520 Q-TOF with an Agilent 1200 LC system. BASL-labeled samples were recorded on an Agilent 1290 Infinity II with an Agilent 6560 Ion Mobility Q-TOF-LC/MS or an Agilent 1200 series with an Agilent 6510 Q-TOF LC/MS. Charges were assigned to peaks in the m/z spectra to allow deconvolution into a neutral mass spectrum using the open source program mMass [44].

### DEER Spectroscopy

DEER experiments were carried out on a pulsed ELEXSYS E580 (9 GHz) spectrometer (Bruker), cooled to 50 K with a continuous-flow helium CF935 cryostat (Oxford Instruments) and an ITC 502 temperature control system (Oxford Instruments). 4-pulse DEER sequence (π/2_*ν*obs_ − *τ*1 − π*ν*_obs_ − *t* − π_*ν*pump_ − (*τ*1 + *τ*2 − t) − π_*ν*obs_ − *τ*2 − *echo*) was applied [3], with π/2_*ν*obs_ pulse length of 16 ns, π_*ν*obs_ pulse length of 32 ns. Pump pulses were applied at the maximum of the field sweep spectrum with the observe pulses 65 MHz lower. τ1 was varied by incrementing the first π_*ν*obs_ pulse position over eight steps of 56 ns for averaging of the deuterium nuclear modulation. Phase-cycling was applied. Matlab based program DEERAnalysis was used to correct for exponential background decay due to intermolecular interactions and to calculate the inter-spin distance distribution [45].

### Labeling Site Simulations

MTSSL labeling simulations to predict accessibility were carried out using the Matlab based program MMM (Multiscale Modeling of Macromolecules), which performs a computational site scan based on a rotamer library approach to provide a prediction of the conformational distribution of spin labels [46, 47]. The statistical partition function (PF) is used as a measure for the tightness of the site, with small values corresponding to large positive interaction energies between label and protein. PF values less than 0.05 indicate difficulties in labeling and are likely to cause a change in protein structure. This work was carried out using crystal structures of HD-PTP_CC_ domain and HD-PTP_Bro1_ domain [38, 48].

### BASL Synthesis

BASL was prepared according to previous reports [36]. Briefly, 4-oxo-2,2,6,6-tetramethyl-1-piperidinyloxy radical (4-oxo-TEMPO) was brominated and converted via Favorskii rearrangement to 4-bromo-3-carboxy-2,2,5,5-tetramethyl-2,5-dihydro-1H-pyrrol-1-yloxyl radical. Thionyl chloride was used to convert this to its acyl chloride before reduction with sodium borohydride to give the alcohol, 4-bromo-3-hydroxymethyl-2,2,5,5-tetramethyl-2,5-dihydro-1H-pyrrol-1-yloxyl radical. This was oxidized with pyridinium dichromate to give the aldehyde, 4-bromo-3-formyl-2,2,5,5-tetramethyl-2,5-dihydro-1H-pyrrol-1-yloxyl radical (BASL).

## Results and Discussion

### BASL Labeling of the Three Cysteine CC Domain

HD-PTP_CC_ contains three cysteines in the wild type sequence: C425, C628, and C697. It also contains twenty-three lysine residues. Our previous work showed that, using MTSSL, all three cysteines were fully labeled by mass spectrometry (Fig. S1), which allowed use of DEER to measure inter-spin distances within the native protein and three single cysteine mutations [38].

**Fig. 2:**
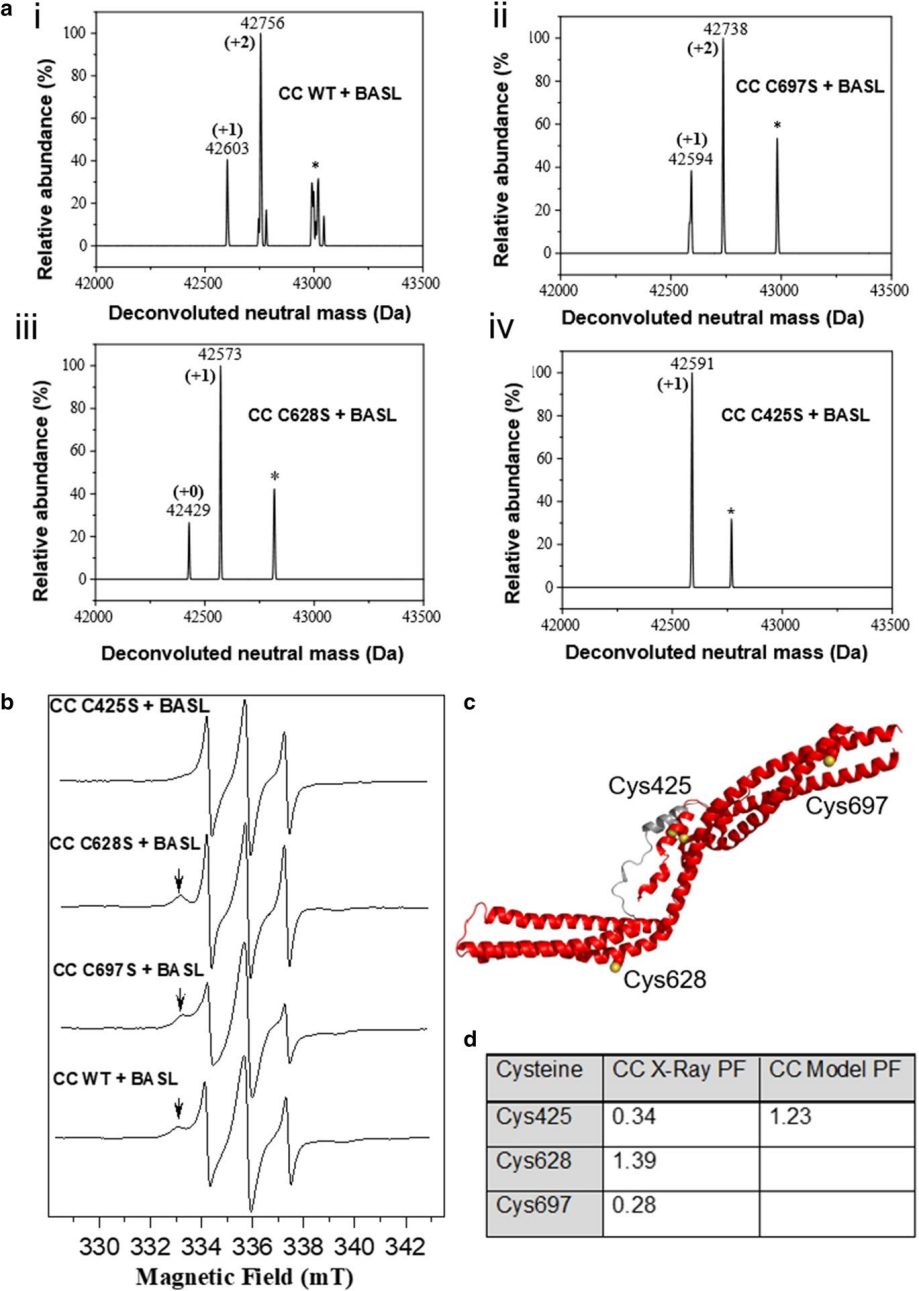
HD-PTP_CC_ labeling. **a** Deconvoluted mass spec of BASL-labeled HD-PTP_CC_ wt. * Marks unidentified peaks. (i) Wildtype and (ii, iii, and iv) mutants. **b** CW EPR spectra of BASL-labeled HD-PTP_CC_ wt and mutants. Arrow marks a peak associated with C425. **c** Cartoon structure of CC domain crystal structure (red) and helix 2 hinge model (gray) with cysteine residues shown as spheres and sulfur atoms in yellow. **d** Partition functions (PF), as a measure of MTSSL labeling site accessibility, calculated for all three HD-PTP_CC_ cysteine residues using the X-ray crystal structure (5LM1), and a second calculation for C425 using a molecular dynamics model

HD-PTP_CC_ wt was labeled with BASL, under the same conditions as MTSSL, which resulted in addition of only two labels by mass spectrometry (Figs. 2ai and S2). To show that BASL was labeling cysteines rather than lysines, and to find out which cysteine was left unlabeled, the same procedure was also carried out on the three cysteine to serine mutants. C697S (Fig. 2aii) gave a major mass spectrometry peak at + 2 BASL labels, like the wild type, indicating that Cys697 was not being labeled. C628S and C425S (Fig. 2aiii and aiv) both had major peaks of + 1 BASL, one less than the wild type, indicating that both these cysteines were labeled in the wild type sample. All the mass spectra contained an additional small peak at around m + 387, highlighted in Fig. 2a with asterisks. The mass difference relative to the unlabeled proteins did not correspond to multiples of 166 for BASL modification but presumably represented a small molecular adduct. CW EPR spectroscopy was carried out on the labeled wild type and mutant HD-PTP_CC_ samples (Fig. 2b). The spectra are multi-component consisting of three sharper resonances overlapping three broader resonances; most likely from different rotamer states representing varying dynamic motion. The spectra are all subtly different, however, differences in the spectra upon mutagenesis can be discerned; for example, the loss of the feature at around 333 mT is present in all spectra except C425S (indicated with an arrow in the figure). Particularly, a fast motion component is present for the 628S mutant and likely represents a population of free spin label.

To rationalize the labeling preference of MTSSL and BASL, cysteine site scans were performed on the HD-PTP_CC_ structure using MMM [46, 47]. This program was employed to superimpose MTSSL rotamers at each cysteine and uses internal and interaction energies to predict the most stable rotamers. Ideally a similar approach would be applied to the BASL label if crystal structures were known of the label and full rotamer simulations calculated. However, here our use of partition function for the MTSSL side chain has given a useful prediction of accessibility. The average PF over the three available HD-PTP_CC_ crystal structures for C628 was 1.39, corresponding to an easily labeled site. C425 and C697 had a PF of 0.34 and 0.28, respectively, indicating similar accessibility for both sites. Our previous work showed that MTSSL labeling combined with DEER was consistent with molecular models of the protein where the central helix is flexible (Fig. 2c). Repeating the MMM site scan using one of the molecular models gave C425 a PF of 1.23, close to the PF for C628. Together these analyses support a steric hindrance argument for why BASL can label C628 and C425 but not C697.

### BASL Labeling of the Six Cysteine Bro1 Domain

**Fig. 3:**
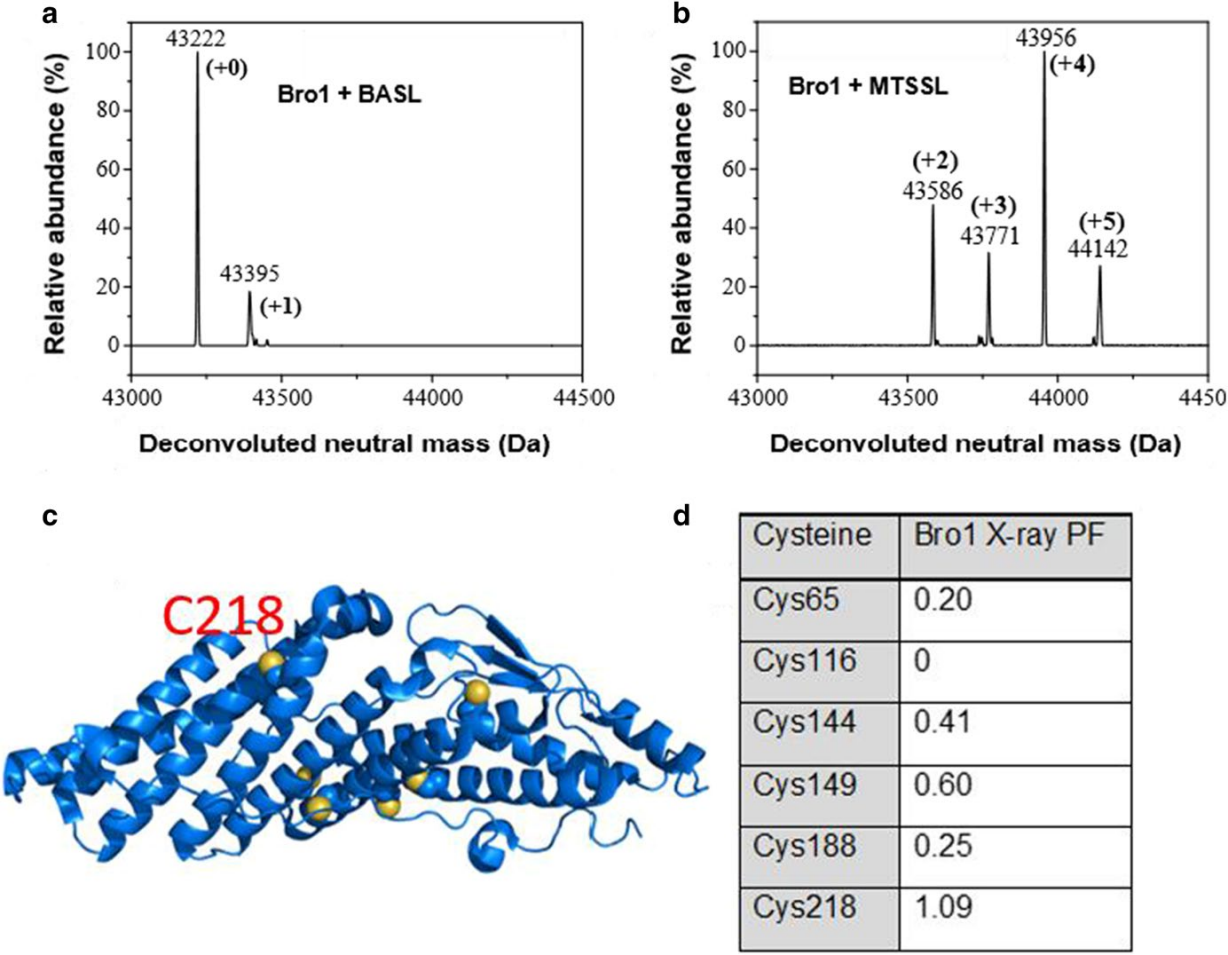
Bro1 domain labeling. **a** Deconvoluted mass spec of BASL-labeled HD-PTP _Bro1_. **b** Deconvoluted mass spec of MTSSL-labeled HD-PTP_Bro1_. **c** Cartoon structure of Bro1 domain crystal structure (blue) with six cysteine residues shown as spheres and sulfur atoms in yellow. C218 is marked. **d** Partition functions (PF), as a measure of MTSSL labeling site accessibility, calculated for all six Bro1 cysteines using the X-ray crystal structure (3RAU)

HD-PTP_Bro1_ has six cysteines (Fig. 3c) which are much more buried than in the CC domain. It also contains twenty-nine lysine residues. Our initial attempts to label with MTSSL resulted in protein precipitation in as little as 1 hour, making the protein unusable for further study. We next tried to use moderate conditions, with MTSSL at a stoichiometric molar ratio (one label: one cysteine). This gave a heterogenous mixture of labeled protein with mainly four labels but also two, three, and five-labeled protein (Fig. 3b), and after excess label removal through size-exclusion chromatography, no further precipitation was observed. The explanation for this seems to be that MTSSL has the potential to label every cysteine in the Bro1 domain but this is avoided when the label excess is reduced, avoiding saturation; the protein is stable having one to five labels but once this reaches six labels it denatures and precipitates.

In contrast, when BASL was added in a 30 × excess this caused no precipitation even after overnight labeling. Mass spectrometry after BASL labeling showed mainly unlabeled protein, with a small contribution of singly labeled protein (Fig. 3a). The presence of a small amount of spin labeled sample was confirmed by CW EPR (See Fig. S3).

The Bro1 domain was also analyzed for MTSSL labeling sites with MMM. Only one cysteine is completely buried (C116, PF = 0), which is consistent with the MTSSL mass spectra showing up to five labels, leaving one cysteine unlabeled. C218 has the highest accessibility (PF = 1.09) with the remaining four cysteines having PFs ranging in the range 0.40–0.60. C218 is highly likely to be the cysteine residue that is labeled to a low extent by BASL, with the others being inaccessible for labeling.

Our results do not support the concern that BASL would label lysines as well as cysteines. HD-PTP has multiple lysine residues in both CC and Bro 1 domains but we found no evidence for lysine labeling. Previously, the reported reaction conditions for cysteamine used acetonitrile solvent in the presence of a base, 1,8-diazabicyclo[5.4.0]undec-7-ene at room temperature [37], whereas the protein labeling was performed at pH 8.5 aqueous buffer at 4℃ which appears to avoid any amine reaction.

### DEER Distance Measurement Using BASL

**Fig. 4:**
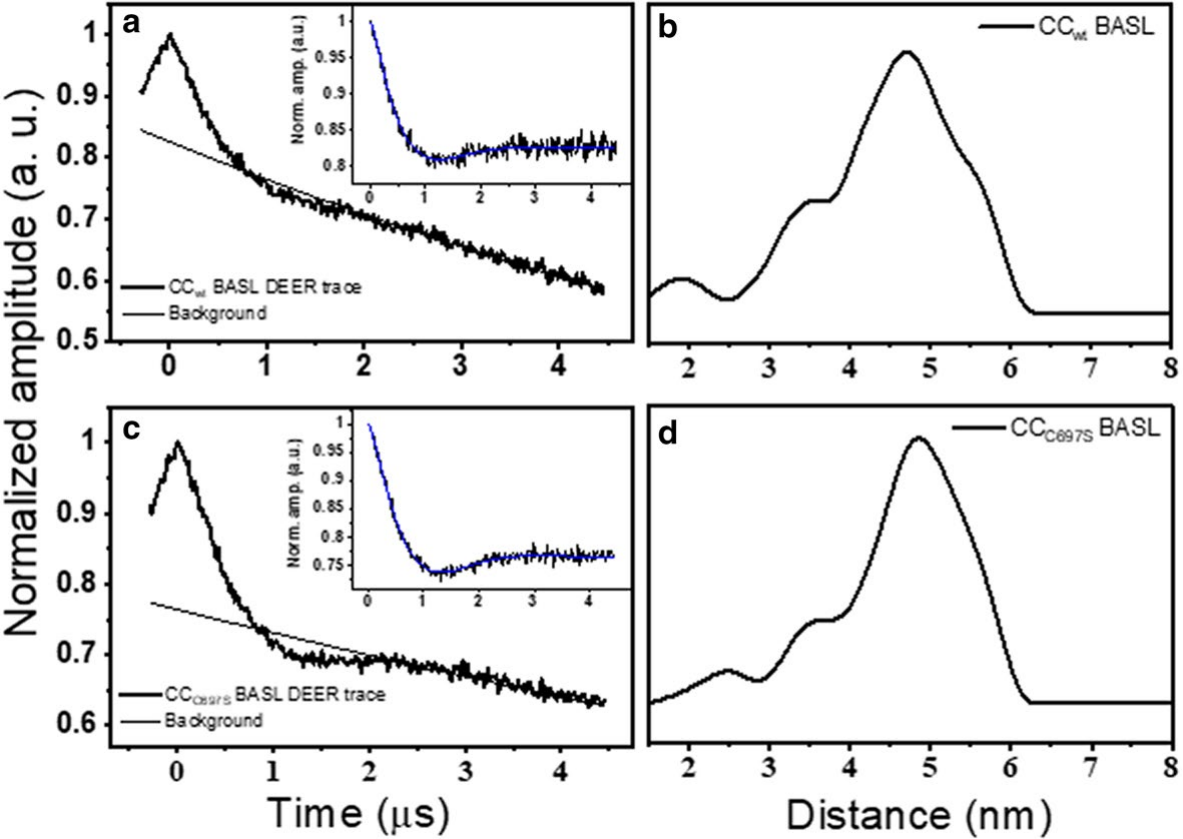
Normalized four-pulse DEER trace of BASL-labeled **a** HD-PTP_CC_ and **c** C697S mutant. Insets: Trace after subtraction of a monoexponential decay. Solid blue line is fit using distance-domain Tikhonov regularization. Resulting distance distributions of **b** HD-PTP_CC_ and **d** C697S mutant

DEER spectra for BASL-labeled WT and C697S CC domain were almost identical (Fig. 4), which corroborates the mass spectrometry and CW EPR evidence that C697 is not labeled by BASL, and, therefore, the distance distribution was between labels attached to C628 and C425 only. The distance distribution for both showed a broad and asymmetric peak with a maximum height at 4.6 nm although no significant narrowing of the distribution width was observed. The corresponding published distance using MTSSL [38] was slightly shorter (4.2 nm) with similar modulation depths. This suggests comparable DEER data can be obtained with BASL compared to MTSSL.

## Conclusions

Previously, DEER was used to study the structure of HD-PTP_cc_ using MTSSL [38]. However, we found ourselves somewhat limited in the scope of this methodology by the number of cysteine residues present in HD-PTP_BRO1_. Initially, the use of MTSSL produced protein precipitation and on lowing the excess gave heterogenous multiple labeling, which would need many more experiments to untangle. The use of BASL as an alternative to MTSSL contributed to diminishing the problem.

BASL was found to be selective for surface cysteines causing the more unfavorable buried positions to be avoided without need for mutagenesis. Of course, mutagenesis of all six Bro1 cysteines and CC C697 could be used to obtain the same labeled HD-PTP samples reported here, but multiple mutations would increase the likelihood of effecting expression or solubility of the resulting recombinant HD-PTP. Using BASL labeling as an alternative to the potentially intensive mutagenesis approach needed for an MTSSL study, we demonstrate how a useful EPR conformational probe can be gained with relatively minimal effort.

A possible drawback of BASL is the presence of the aldehyde group adding to the conformational flexibility and coordinability. It is also a source of reactivity which could be a hindrance depending on the chemical conditions but could be further exploited to examine further bound structures. The three rotatable bond tether of BASL, although not demonstrated here, may also prove to be a potential advantage minimizing structural perturbance of protein structures and complexes. A further potential advantage is that BASL labeling does not use a labile disulfide bridge, which for MTSSL has been shown to cleave inside cells [49].

The field of new spin labels used to provide distance constraints on proteins continues to grow [29–33] and will no doubt increase the use of EPR for biological studies. Further investigation of BASL on a greater array of proteins will be needed to better predict reactivity and to help gain structural information needed to predict rotamers for DEER distance distributions. Since MTSSL seems to have moderate selectivity against buried cysteines whereas BASL is far more selective, the investigation of this property using a derivative of BASL with a different Michael acceptor could be investigated. Ongoing work investigates the use of BASL versus MTSSL to show HD-PTP_CC_ domain binding to its protein partners.

## Supplementary Information

Below is the link to the electronic supplementary material.

Supplementary file1 (DOCX 937 kb)
