# Supplementary material for: Spin Labeling of Surface Cysteines Using a Bromoacrylaldehyde Spin Label
Source: Appl Magn Reson. 2021 Jun 10;52(8):959–70. doi: 10.1007/s00723-021-01350-1 (PMC8550513; doi:10.1007/s00723-021-01350-1)
Supplement: Supplementary file 1 — Supplementary file1 (DOCX 937 kb) [file 723_2021_1350_MOESM1_ESM.docx]

Supporting Information

**Spin labelling of surface cysteines using a bromoacrylaldehyde spin label**

Graham Heaven^1^, Michael A. Hollas^1^, Lydia Tabernero^2^ and Alistair J. Fielding^3,*^

^1^ Department of Chemistry, The University of Manchester, Manchester M13 9PL, UK.

^2^ School of Biological Sciences, Faculty of Biology Medicine and Health, University of Manchester, Manchester, M13 9PL, UK.

^3^ Centre for Natural Products Discovery, School of Pharmacy and Biomolecular Sciences, Liverpool John Moores University, Liverpool L3 3AF, UK.


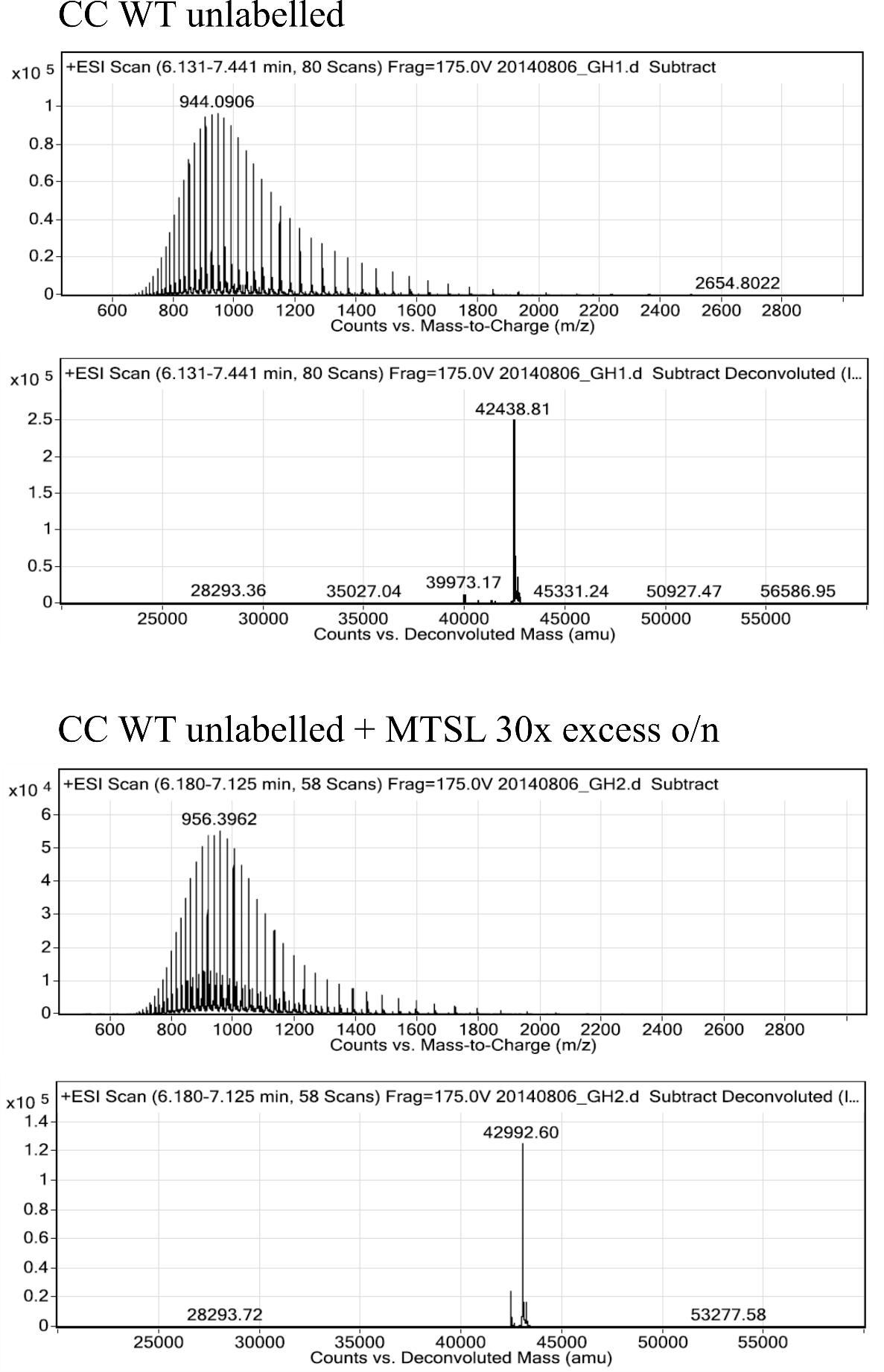


**Figure S1.** Mass spec of HD-PTP CC wt labelling with MTSSL.


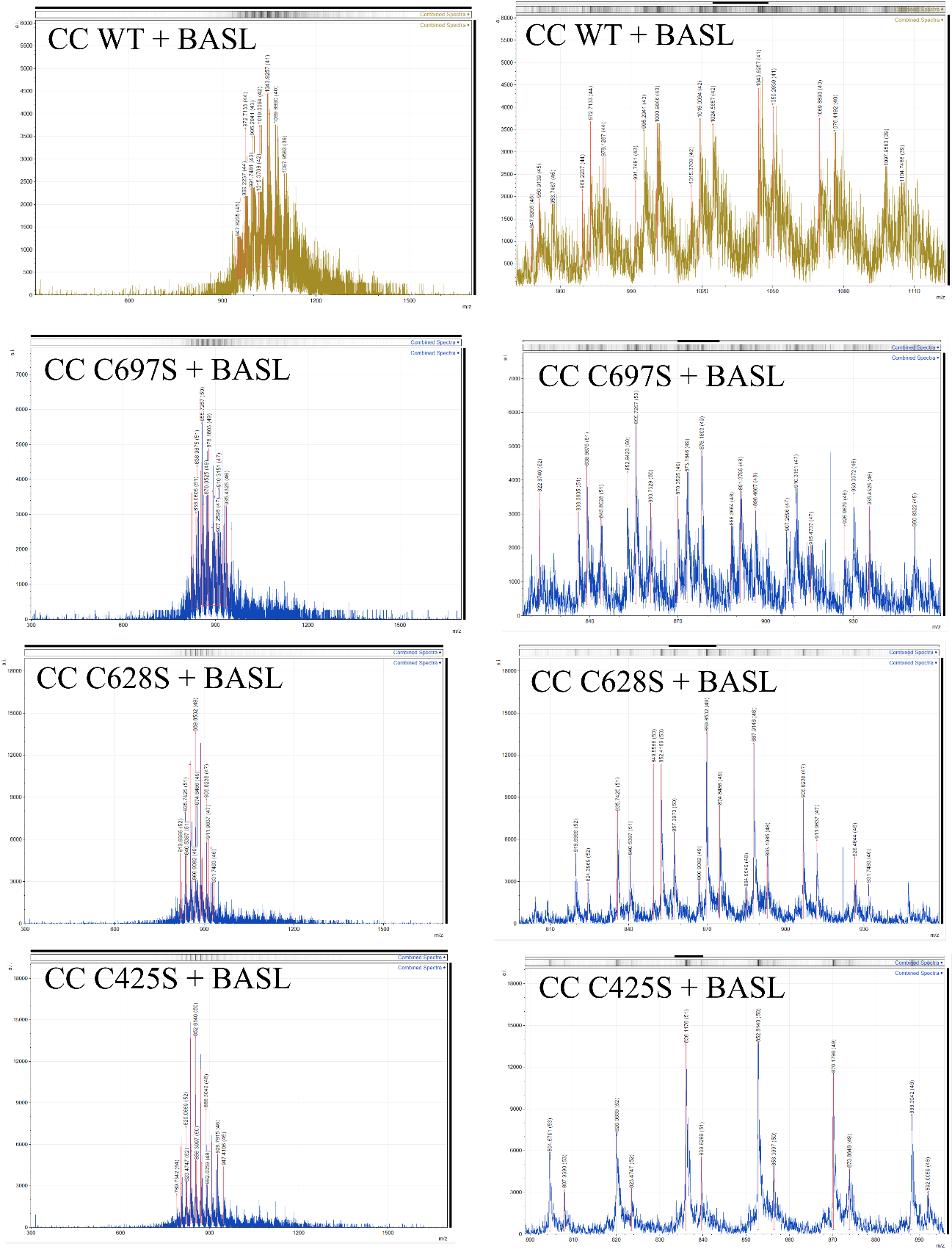


**Figure S2.** Raw ESI mass spec of CC BASL-labelled samples.

**Figure S3**. CW EPR spectrum of BASL-labelled HD-PTP Bro1. * Marks the presence of the label in a background of free label.
